# Iodoform in Articular Tuberculosis

**Published:** 1890-03-08

**Authors:** 


### IODOFORM IN ARTICULAR TUBERCULOSIS.

Since the tubercular, *i.e.*, bacillary nature of all "scrofulous" diseases of the joints has been fully recognized, the value of iodoform as a local remedy has been admitted on all hands. But the question has been, and is still, how to apply it, especially when there is no breach of surface, whether as the result of operation, or of the spontaneous opening of collections of pus; or, apart from matters of detail, whether to favour or to impede the *absorption* of the drug.

At Bonn, Trendelenburg originally used injections of five per cent. iodoform ether, but soon found himself compelled to desist on account of the severe pain and inflammatory disturbance set up, occasionally ending in sloughing or gangrene. But since he has substituted freshly prepared iodoform oil, 1:5, he has met with no unpleasant results. The injections of 2-3ccms. (m XXX-L) are repeated every eight days under the strictest antiseptic precautions, and are made into the swollen tissues. Sometimes the favourable influence is perceptible after three or four injections, at others not till many more, but when once the joint begins to improve the subsequent progress is rapid. Fistulæ previously existing give much trouble. The question of rest or exercise of the joint depends, he says, on circumstances.

Volkmann himself, as we learn from an excellent report of his practice by an old pupil and assistant, was averse to solutions of iodoform, aiming at restricting the action of the drug to the actual seat of the disease. He used an emulsion of iodoform, in the finest powder, 5; mucil, gum. arab., 23; glycerine, 83; aq. distill. ad 500. This gives a ten per cent. suspension, but no solution whatever, so that when the fluids are absorbed the iodoform remains *in situ* to act on the bacilli. He made his injections in all cases into the capsule of the joint, of course under the strictest antiseptic precautions, whether abscesses had formed or not. The injections, consisting of 5—30 or 20—80 drops, according to the severity of the disease, were mostly repeated four times a week.

During the year and a-half preceding his death Volkmann had carried out this treatment in forty-three cases, all of the gravest character and giving no chance of recovery except in excision; in half of them there were "cold" abscesses, but in none had any spontaneous evacuation occurred or operation been attempted. Every one recovered more or less completely the usefulness of the limb, and absolutely as regards the actual disease. Pain, sharp but short, sometimes followed the injection, and occasionally a slight elevation of temperature. But in nearly all the pain previously present subsided or disappeared with surprising rapidity, the swelling of the joint and soft parts more gradually, and abscesses not entirely until after repeated injection. He generally employed passive movements or massage of the limb after the completion of the injection, maintaining absolute rest or fixity only in exceptional cases.

